# Adapting Traditional Healing Values and Beliefs into Therapeutic Cultural Environments for Health and Well-Being

**DOI:** 10.3390/ijerph19010426

**Published:** 2021-12-31

**Authors:** Bruno Marques, Claire Freeman, Lyn Carter

**Affiliations:** 1 Wellington School of Architecture, Te Herenga Waka—Victoria University of Wellington, Wellington 6012, New Zealand; 2 School of Geography, University of Otago, Dunedin 9016, New Zealand; cf@geography.otago.ac.nz; 3 Te Tumu School of Māori, Pacific Island and Indigenous Studies, University of Otago, Dunedin 9016, New Zealand; lynette.carter@otago.ac.nz

**Keywords:** therapeutic landscapes, therapeutic environments, Indigenous knowledge, Mātauranga Māori, Rongoā Māori, traditional healing, health and well-being, cultural landscapes, cultural geography, landscape architecture

## Abstract

Although research has long established that interaction with the natural environment is associated with better overall health and well-being outcomes, the Western model mainly focuses on treating symptoms. In Aotearoa/New Zealand, the Indigenous Māori have long demonstrated significantly more negative health outcomes than non-Māori. Little research has examined the causes compared to Western populations or the role of the natural environment in health outcomes for Māori. An exploration of rongoā Māori (traditional healing system) was conducted to ascertain the importance of landscape in the process of healing. Eight rongoā healers or practitioners took part in semi-structured narrative interviews from June to November 2020. Transcribed interviews were analysed using an interpretative phenomenological analysis and Kaupapa Māori techniques. The findings show how rongoā is underpinned by a complex set of cultural values and beliefs, drawing from the connection to wairua (spirit), tinana (body), tikanga and whakaora (customs and healing), rākau (plants), whenua (landscape) and whānau (family). Incorporating such constructs into the landscape can foster our understanding of health and well-being and its implications for conceptualising therapeutic environments and a culturally appropriate model of care for Māori and non-Māori communities.

## 1. Introduction

Indigenous cultures embrace traditional medicine as a preferred form of healthcare in many parts of the world as it holds a more holistic approach to healing, including the interconnection of mind, body, and spirit in a specific context. For centuries, traditional medicine was the only approach to health and illness across different cultures [1,2,3]. Traditional medical systems focus on the relationship between spirituality, healing, illness and landscape [4]. Such systems are deeply influenced by history, environment, places, attitudes, philosophy and traditional healing practices [5]. Scholars have attempted to investigate and characterise traditional medical systems through different health models and theories [6,7], healing approaches [8,9,10] and alternative therapies and treatments [11,12,13]. In addition, many theories and traditional healing practices have been researched based on traditional Chinese philosophies [14], native American healing [15,16], Australian aboriginal practices [17,18], and Indigenous Māori of Aotearoa/New Zealand [19,20]. Although a wide range of views and opinions from Indigenous peoples were reported across these studies, some commonalities were identified. Many studies have identified a set of health practices and approaches embedded in the knowledge and beliefs of a cultural group, incorporating plant, animal and/or mineral-based medicines, spiritual techniques, healing techniques and exercises, often transmitted orally from generation to generation with the intent to solve health problems and maintain well-being [21,22]. However, in contrast, Western-based medicine places little emphasis is placed on cultural values and beliefs associated with healing and relegates the importance of place and landscape.

The holistic nature of a Māori view of health contributes to understanding the interrelationship of mind, body, spirit, and place as the basic tenets of life [23]. For the Indigenous people of Aotearoa/New Zealand, their relationship to the natural environment, both physical and spiritual, is the core of their health and well-being. The medicinal properties of Aotearoa/New Zealand’s native plants have been used historically by Māori in the form of Rongoā Māori (traditional Māori healing), which continues to this day. Rongoā Māori (RM) is a holistic system of healing derived from Māori philosophy and customs [24,25] that has faced many challenges with colonisation, the Tohunga Suppression Act of 1907, for example, that aimed at replacing tohunga (traditional Māori healers) with Western medicine and Western trained medical practitioners. The effects of globalisation and urbanisation have put Māori cultural knowledge and traditions at risk of disappearing. However, a growing interest in traditional Māori medicine and Indigenous knowledge in the context of health and well-being is clearly noticed, leading to varying patterns of integration with mainstream medicine.

While the mainstream Western medical health system dominates in Aotearoa/New Zealand, it is often criticised for its reductionist and biological approach to health treatment based on quantifiable outcomes [26,27]. As such, there is little room to encompass Māori cultural concepts such as holism, the impact of the whānau (family) and wairuatanga (spirituality), all intrinsic to Māori health and well-being. Traditional healing or traditional medicine has been practised to some degree in all cultures and societies to maintain health and treat disease or illness. This paper explores the views and contributions of Māori healers and seeks to ascertain their understandings and perceptions of RM. It aims to provide a valuable exploration of the cultural values and beliefs that support healing, health and well-being and the importance of landscape.

## 2. Rongoā Māori

While there are opposing views about the degree of effectiveness of traditional healing practices in the modern world, some of the positive contributions of traditional medicine are indisputable. Traditional medicine attempts to understand the person’s health and well-being by situating it within the surrounding sociocultural and natural environments and treating the person as a unified whole. According to Senanayake [28], Indigenous therapeutic practice is interdisciplinary, combining knowledge of botany, toxicology, chemical physics, biochemistry, and psychology, making it deeply grounded in the surrounding landscape. Landscapes hold great importance in individual health and well-being as well as the communal quality of life. Indigenous health is interdependent on ecosystems and the services that nature provides, emphasising that it is through the connection to the landscape and ecosystems that well-being is achieved [29]. In this context, RM is a system of healing techniques and practices embedded in the cultural history of Māori. As a holistic approach, RM encompasses physical, cultural customs and beliefs, and spiritual methodologies passed down through generations [30].

**Figure 1:**
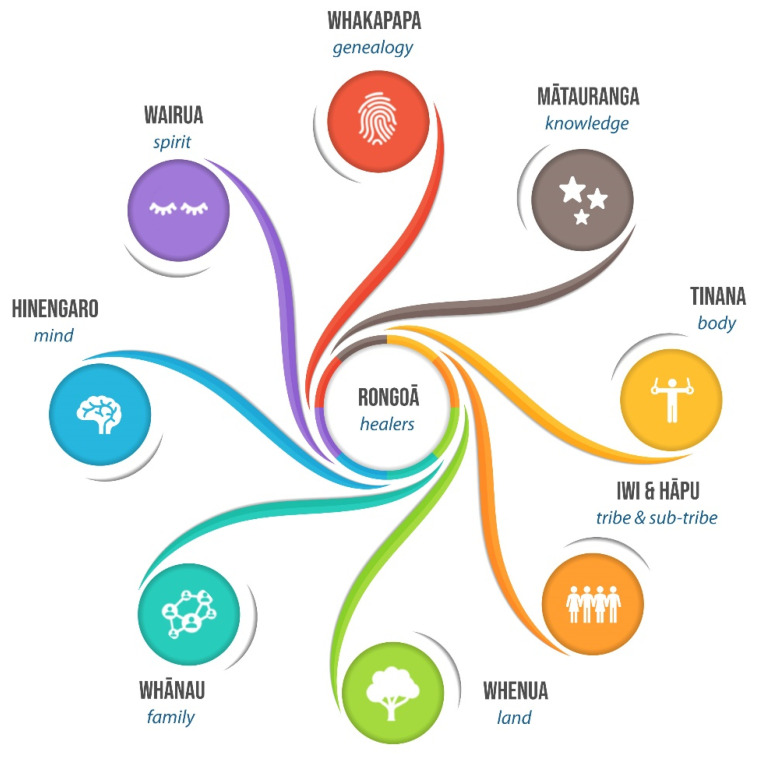
The essential elements that are part of the practice of rongoā.

An essential aspect of RM and its practices is the locally specific nature of healers and the connection to place, their whenua (landscape), iwi (tribe), hapū (sub-tribe) and whānau (extended family). While many researchers stressed the importance of the concept of place [31,32,33,34,35], few studies mention the importance of self, body, place and landscape [36,37,38]. Central to this understanding, three dimensions of Māori knowledge—whakapapa (genealogy) as a way of knowing things, understandings of time, and the importance of the spoken word rather than visual representation—are used to demonstrate how Māori identify themselves, conceptualise the body as an arbiter of interaction with the environment, and create landscape through place naming. The world is represented through oral narratives, tales, songs, customs, place naming, and rituals that impress ancestors and deities into the landscape so that a place and its knowledge cannot be separated. This forms the base of mātauranga Māori (Māori knowledge), developed over thousands of years based on the intricate, holistic and interconnected relationship with the natural world [29], recognising as well the significance of intangible values and how landscapes shape cultural identity [39]. Māori cultural perspectives are deeply rooted in views of hinengaro (mind), tinana (body) and wairua (spirit), which is vital to rongoā healers [25], who need to access whakapapa (genealogy), whānau (extended family) and whenua (land) as essential aspects for healing a person’s health (Figure 1). The complex intersection of people, place and ‘nature–culture’ relations highlights the dimensions of Indigenous life and how that affects health and well-being [40].

For Māori, a person’s health and well-being is inextricably interconnected with the health of the ecological surroundings and emotional ties with others, emphasising the importance of maintaining balance and harmony with nature. McGowan [41] believes that this relationship with nature is better observed in forests to learn about the natural environment and our place within it. Māori believe that mauri (life force) runs through everything, connecting us to our natural environment, described as taha wairua or the spiritual side or dimension [41]. Interactions with divine, spiritual, and ancestral beings that are part of the natural realm foster the diagnosis of an illness through the surrounding landscape. However, this spirituality does not correspond directly to a Western understanding of spirituality; rather, it is seen as a world of connections that flows through that wholeness. As we are only one small part of a far larger ecosystem, Māori deem that humanity must maintain the health of our surroundings, and in turn, the natural equilibrium of all things [40]. This connection to whenua (land) brings forth the importance of care within the Māori value system, which calls for humans to be kaitiaki (caretakers) of the mauri (life force) in each other and in nature [42]. If we are in harmony with ourselves and our surroundings, then we are well [41].

According to te ao Māori (Māori worldview), plants and humans are the offspring of Tānē Mahuta, god of the forests. However, plants were created before humanity, giving them a senior status [30]. As elder relatives, plants are seen as a link between humans and the sacred ancestors of Papatūānuku (Mother Earth) and Ranginui (Sky Father) [43]. Māori used their extensive plant knowledge to harvest and utilise the resources that Aotearoa/New Zealand’s unique ecological systems offer. However, this relationship between humans and nature was always one of kaitiakitanga (guardianship) and aroha (love and respect) [41]. The benefits of this relationship had to be reciprocal. Māori understood that the best way to take care of themselves was to take care of the natural world so that it could continue to support them and their children for generations [44]. For instance, the incorporation of wairuatanga (spirituality), often in the form of karakia (chants), ensures that appropriate rituals and traditions are upheld before, during and after the healing process is conducted to maintain the balance between living and non-living things (McGowan, 2000). The importance of wairuatanga (spirituality) also explains the need for spiritual rituals before the collection and use of herbal materials [43], another essential aspect of RM.

**Figure 2:**
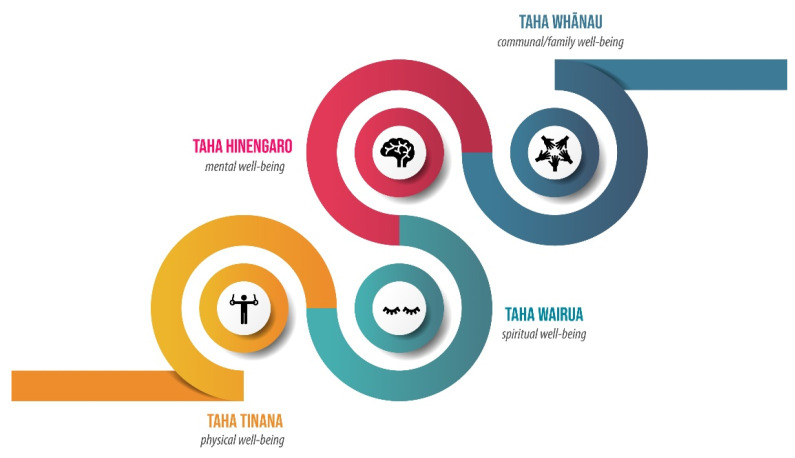
Fundamental aspects of Māori health and wellbeing.

RM is understood today to encompass a wide range of treatments. These can include mirimiri (massage), karakia (chants), wai (water), ritenga (rituals) and rongoā rākau (herbal medicines) [45]. Historically, herbal remedies were applied through various methods, including poultices, steam baths, tonics, and teas [46]. These treatments were used for numerous ailments and complaints both internally and externally by harvesting the leaves, sap, bark or roots from certain plants [20,47]. Currently, RM focuses on treating the illness at the source or on prevention entirely, rather than simply seeking to remedy the symptoms once they become evident [48]. The emphasis is placed on strengthening family relationships and mental health as these elements are fundamental to a person’s well-being and must be considered in conjunction for a patient to heal properly [25,41]. Health and well-being, as intertwined constructs, result from a subtle balance between mind, body and spirit [49]. In this way, the treatment differs in its principle values from Western medicine. While the Western model concentrates on treating the symptoms, RM aims to heal by improving the mental, physical and spiritual well-being of a patient to generate awareness about maintaining a healthy equilibrium [50] (Figure 2). With rongoā practices, effectiveness is not simply related to the chemical composition and pharmacological action of a plant, but mainly to the process that is conducted within the context of traditional healing [46].

In summary, RM is strongly associated with concepts of health and well-being. Such practices permit alleviation of symptoms and enhance well-being for individuals and the collective while promoting cultural traditions and values associated with place and whenua (land), and safeguarding environmental stewardship for iwi (tribe), hapū (sub-tribe) and whānau (family). The current challenges of rongoā practices are in tune with the changes we face in our environment and society, where difficulties are noticed in retaining local flora and adapting to the existing health sector [24,51]. Despite that, an increase in Māori healing practices has been recorded [30,48], supporting the value of traditional ecological knowledge, giving indigenous, holistic understandings and approaches a new-found contemporary significance.

## 3. Materials and Methods

**Figure 3:**
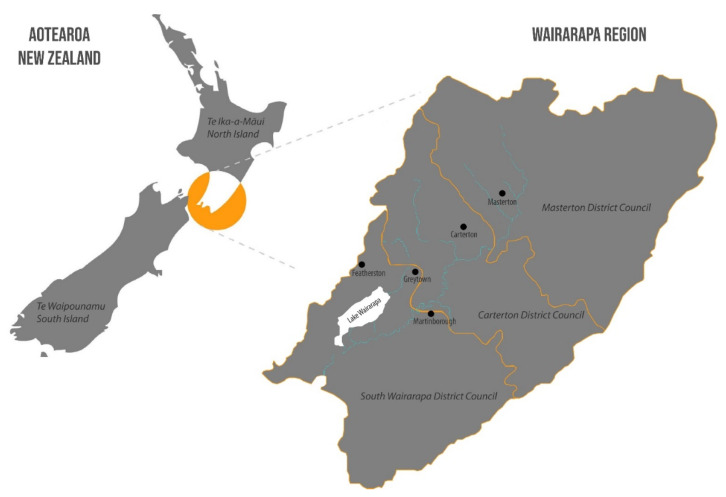
In the context of Aotearoa/New Zealand, the Wairarapa region has three district council boundaries: South Wairarapa District Council, Carterton District Council, and Masterton District Council. Geographic coordinates 41.2676° S, 175.3550° E.

This study is situated in the Wairarapa region of Aotearoa/New Zealand’s North Island (Figure 3). Located in the south-eastern corner, 80 km northeast of the capital city of Wellington, the Wairarapa region is known for its sheep grazing and dairy farming. It has a long history of occupation by Māori tribes, namely Ngāti Kahungunu ki Wairarapa and Rangitāne o Wairarapa. The regional population steadily increased throughout the twentieth century, reaching 41,097 individuals in 2013 [52]. The Wairarapa region has a total area of 5900 km^2^ (or 590,000 hectares), but the rohe (tribal boundaries) of both Rangitāne o Wairarapa and Ngāti Kahungunu ki Wairarapa extends to more than 10,000 km^2^ (one million hectares) to include the north-adjoining Hawke’s Bay region [52]. From the recorded population, 51.5% are females (or 21,162 individuals) and 48.5% are males (or 19,956 individuals). Looking at the population distribution, the higher proportion of people are in the younger age groups (0 to 17 years) and the older age groups (50+ years), representing jointly 65% or 26,832 individuals. Concerning the major ethnic groups, 87% of the region’s population identify as of European ancestry (or Pākehā), 17.5% as Māori and a smaller proportion of people have identified as Pasifika. Although the Māori population increased by over 15% between 2006 and 2013, this ethnic group represents 7173 individuals of the Wairarapa region’s total population.

### 3.1. Process

The methodology used in this study incorporates mātauranga and tikanga Māori (Māori knowledge and customs) but also includes aspects of Western research methodology such as a literature review, recorded and transcribed semi-structured narrative interviews (following a wānanga approach of meeting and discussion) and analysis using an interpretive phenomenological approach (IPA). To establish a collaborative partnership with Māori participants, a kaumātua (elder) of Ngāti Kahungunu ki Wairarapa iwi (tribe) and another from Rangitāne o Wairarapa iwi (tribe) worked together with the researcher. Both kaumātua (elders) have a long association with the researcher, which emphasises the importance of whakapapa (genealogy) and whakawhanaungatanga (to establish and maintain relationships). However, more importantly, both supported the nature of the research and assured that Māori cultural protocols were followed. For instance, the use of koha (gift), where a small gift and food was given to each participant to thank them for their time, was adopted. The study development included consultation with Te Whare Wānanga o Otāgo—University of Otago’s Ngāi Tahu Research Consultation Committee, whose members review research proposals involving research with Māori in Aotearoa/New Zealand. The study was reviewed and approved by the Human Ethics Committee of the Te Whare Wānanga o Otāgo—University of Otago and conducted within their ethical guidelines. All participants gave full informed consent.

### 3.2. Recruitment

Snowballing or chain referral techniques were employed to recruit rongoā healers as it allows for a study sample through referrals among people who share or know of others who have similar characteristics of importance to the research [53]. Participation in this study was open to any that self-identified as Māori healers or rongoā practitioners. Working with established Māori elderly health groups and through the general hapū/iwi (sub-tribe/tribe) was found to be an effective approach to recruit participants. Two participants were known to the researcher, while the remaining participants were identified by friends’ and associates’ contacts. Information sheets were provided at the start of each interview. Eight interviews were conducted, where 75% were wāhine (women) and 25% were tāne (men). The age groups were between 30–40 (12.5%), 40–50 (25%), 50–60 (37.5%) and 60+ (25%). Historically, each iwi (tribe) may have different perspectives and knowledge, and hence, interviewing mana whenua (those who have ancestral relationships with a particular territory) within their rohe (tribal boundaries) would be the best practice [40]. However, over 85% of Māori now do not live in their rohe [54], so they are not mana whenua; instead, they are termed ngā matāwaka or taura (those who are settled in a different territory to where their tribal ancestors settled). From the 8 participants, 63% were mana whenua, while the remaining were ngā matāwaka with mixed whakapapa (genealogy). Participants practised a wide range of healing techniques within Rongoā Māori.

### 3.3. Interviews

Data collection took place from June to November 2020. Face-to-face semi-structured narrative interviews took place in three different marae (Māori meeting places), namely, Papawai Marae (Greytown), Hurunui-o-Rangi Marae (Gladstone) and Te Rangimarie Marae (Masterton), the latter having a Māori health clinic on-site. The interviews were used to allow multiple topics and concepts to be explored in an open-forum format [55]. A questionnaire with 8 open-ended questions and 8 dichotomous (yes or no) questions was generated to guide the interviews. Participants were introduced to the aim of this research in understanding the connection between landscape and health and well-being through the importance of Rongoā healing practices. The questionnaire was adapted after testing and discussion with Māori elders as part of an early pilot study. The questions covered healing practices, outdoor environments, future use and protection and general demographics (Table 1). Interviews started with a pepeha (introduction) to establish the relationship between people that at that time did not have a bond and become part of the whenua (land) and of the mana whenua (those who have ancestral relationships with a particular territory). Interviews lasted between 45 and 60 min and were audio-recorded.

Research is often viewed with doubt by Māori and has been implicated in the process of colonisation. Māori-led research has, in part, grown out of dissatisfaction with prevailing methodologies [56]. In recognition of this background, a kaupapa Māori approach was adopted with the intent to make a positive change [57]. It is necessary to acknowledge that the lead investigator in this research has no Māori heritage but has a pre-existing relationship established over time with both local iwi (tribe) in the Wairarapa region. Further to that, authenticity and accuracy were secured through engagement with Māori academics and related professionals in the community of practice [58], and, as a result, this research was peer reviewed by the Indigenous groups represented. Therefore, the knowledge gained must be returned for the benefit of those taking part and for the kaupapa, or topic of the research. This acknowledgement of sharing of knowledge was included in the information for participants and was discussed in some detail with the participants before the interviews.

### 3.4. Data Analysis

All interviews were transcribed verbatim and analysed using interpretative phenomenological analytic techniques. This method permits the participants to explore and extract meaningful inferences based on their personal world [59] as its emphasis is on hermeneutics, experiences and in-depth analysis. An essential aspect of the IPA analysis process is the interpretation by the researcher (of the interpretation provided by the participants) of their experiences. The IPA coding provides for a detailed line by line analysis of the experiences and interpretations of each participant, and the identification of themes and super-themes, while observing context, language used and content. This was initially undertaken within each transcript of the interviews by using a qualitative data analysis computer software package, called Nvivo, version 12 [55].

In addition, pūrākau (narratives, stories) was adopted as a method or lens of analysis [60]. This method takes a traditional form of Māori narrative, drawing from and responding to the broader historical, social and political research contexts [60,61]. Through pūrākau (narratives, stories), Māori maintain connections to the land, such as through narratives about landmarks as ancestors of spiritual significance (e.g., a maunga, or mountain, that is tīpuna, or ancestral, to a particular iwi or tribe, who has significant mana, or authority, and thereby confers mana upon his descendants). As a methodology, it assists the researchers in unpacking the wisdom contained in sacred landscapes by the ancestors and draws on kaupapa Māori principles and values to understand and interpret such narratives.

As kaupapa Māori research, emphasis was placed on the mātauranga (knowledge) passed between generations [62] and participants’ experiences as the holders of this knowledge. In addition, care was taken with making connections with participants with the objective that this should accord with tikanga (protocols) [63]. For instance, participants were generally located through iwi (tribe) or personal contacts of the researchers. Interviews were generally undertaken within the mantle of the hapū or sub-tribe, often commenced with a mihi whakatau (official welcome speech), which included karakia (chants), followed by a process of whakawhanaungatanga (to establish and maintain relationships) with food sharing, and concluded with karakia (chants). The essential aspect of karakia (chants) was often reaffirmed in interviews. Its role was to ‘clear the path’ and provide protection for those taking part and ‘lift the heaviness of those discussions’ at the end [62,64]. As part of kaupapa Māori methodology, the principles and practice of hapū (sub-tribe), and the culture, beliefs, language, pepeha (way of introducing yourself) and mātauranga (knowledge) of participants were recognised for the leading role they provided in this research. Te reo Māori (Māori language) was part of the research, and although the interviews were generally conducted in English, participants were free to use either language.

The data analysis adopted a six-step methodology (Table 2) [65,66,67]. It started with the first three stages of the process by coding transcripts, adding first-order themes and subsequently moving coded data to second-order themes, allowing consolidation and data reduction. The fourth stage included clustering groups of themes into a coherent thematic framework. Each step of this methodology involved revisiting earlier stages and re-examining how and why themes were grouped together to ensure consistency between codes and themes, especially in lower-order themes, to avoid premature grouping. Each major decision and stage of the study was discussed in detail by the authors and justified using previous literature. An audit process was used to document each step as well as personal considerations and impacts. Findings were assessed in terms of relevance to current and future theory and practice.

Both research methods help to enable a rich interpretation of the qualitative information provided by participants. The methods, one from Mātauranga Māori and the other from Western psychological research, align as both hold context to be an important factor, and both have been adopted in health research. Kingi [68] refers to this process as a means of facilitating the development of a conceptual framework, while Holloway and Todres [69] defend that ‘thematicising’ is a form of qualitative inquiry that can be conducted with several theoretical frameworks, including kaupapa Māori as reported by Ihimaera [70]. Based on a te ao Māori (traditional Māori worldview), four fundamental elements of health, namely, taha hinengaro (psychological), taha wairua (spiritual), taha tinana (physical) and taha Whānau (family), are used to help clustering groups of themes [71].

## 4. Results

As the data were reviewed and coded, five superordinate overarching, overlapping and interconnected themes related to the theory and practice of Rongoā Māori from a healer’s perspective were identified in the interview transcripts, namely, wairua (spirit), tinana (body), tikanga and whakaora (customs and healing), rākau (plant remedies) and whenua (land) and whānau (family). Each superordinate theme was portrayed in the transcripts with equal importance and is described below using illustrative quotes from participants.

### 4.1. Wairua (Spirit)

Central to rongoā healers is wairua (spirit), mentioned by most as an essential aspect of good health. Despite the difficulty in defining wairua in te ao Māori (Māori worldview) [20], some participants established the connection with the natural order of things, particularly with one’s mauri (life force) or sacredness, acknowledging that the same life force is present amongst all other things. Others mentioned the importance of wairua when communicating with their tīpuna (ancestors) during a healing session.

*“It’s our tīpuna [ancestors] that do all the work…. we are just the vessel.”*

(participant 4, female)

*“When healing, we connect to the whaiora’s [patient’s] ancestors to do the mahi [work].”*

(participant 1, male)

For most rongoā practitioners, wairua was interpreted beyond a life force that is interwoven and connected with everything [6] and instead was acknowledged to influence physical well-being. In addition, healers agreed that healing begins with an assessment of spiritual experiences and understandings. The spiritual connection was fundamental for the healing process as the healers’ views were that the ancestors were responsible for the actual healing. This aligns with the Māori construct that humans are composed of tinana (body), wairua (spirit) and mauri (life force) [30,72], and healing energy originates from the spiritual level and moves through the healer’s body and is then sent to the client [23,51]. All rongoā practitioners accepted that illness comes when mind, body and life force are not connected.

*“If the balance is uneven, and you don’t have that out there in the environment, then you bring that balance back. You must do one thing before you even attempt to go in and start doing.”*

(participant 7, female)

The vast majority of the participants established that a healthy wairua is directly connected to the surrounding environment, including the natural environment (land, mountains, rivers, sea, etc.) and the social environment (family, sub-tribe, and tribe). Through that healthy environment, healers can tap into or connect with the surrounding energy, restoring balance, harmony, and connectedness between the physical, psychological, and spiritual realms.

“*Go into the ngāhere [forests] to get rongoā, you must first ask Tānē Mahuta [god of the forests and birds]. You must go through all those Atua [gods], like Tangaroa [god of the ocean], Tūmatauenga [god of war], Rūaumoko [god of earthquakes, volcanoes and seasons]. You go through the whole lot where ever it is that you have to go....”*

(participant 2, male)

*“Rongoā is like a journey… it involves opening up to the mauri [life force] around you and allow that to come through you…. if your surrounding taiao [environment] is polluted, the healing is not going to work.”*

(participant 5, female)

Most participants agreed that wairuatanga (spirituality) plays an essential aspect in health and wellbeing. That spiritual health is related to the connection with tīpuna (ancestors) and the surrounding mauri (life force) that runs through the existing ecosystems that form part of the whenua (landscape). “Ka ora te whenua, ka ora te tāngata”, if the land is healthy, people are healthy.

### 4.2. Tinana (Body)

For most rongoā healers, tinana (body) was an essential aspect of healing and well-being. The body was not merely seen as tissue and muscle but as the physical systems that impact and be impacted by the psychological/spiritual/social systems that influence overall well-being. Tinana (the physical dimension) is concerned with the human body’s well-being and how the body interacts with the external world [73]. Like the previous superordinate theme, most healers accepted that illness is the physical manifestation of the disconnect between mind, body and life force. Rongoā practitioners defended that the link between mind and body is through emotions. Most believe that negative emotions are held, trapped, or stored in the body and manifest as disease, including the mental and spiritual layers. Such negative emotions can be the catalyst to problems in specific parts of the body. Many defended that emotions are the link between mind and body:

*“If those bad emotions and thoughts are trapped in your body, they can cause damage… it’s all to do with the connection of mind, body and spirit.”*

(participant 1, male)

“*It is when our hinengaro [mind], tinana [body] and wairua [spirit] are separated that disharmony and illness appear in our lives. That can take many forms, from unhappiness to physical malfunction.”*

(participant 3, female)

In addition, some healers mentioned the importance of the body as having an inherent awareness of its own, a power to achieve balance when in disharmony. Other participants mentioned the power of karakia (chants) to heal the body and make sure Māori rituals and traditions are upheld before, during, and after healing.

*“Our tinana [body] is stronger than you think, it has the power to heal itself.”*

(participant 6, female)

*“And with the karakia [chants], you put in that karakia what you need to be done for the tinana [body], for the mauri [life force], for the mana [authority or prestige] of your people. If the balance is uneven, then you must bring that balance back.”*

(participant 4, female)

These descriptions suggest that the body is self-aware and has an intrinsic will to restore harmony and balance [30]. For most healers, that is accomplished by the body’s instinctive urge derived from internal wisdom that fosters the healing potential. In addition, healers emphasised the importance of rituals and traditions, such as karakia (chants), to maintain the connection between mind, body and life force or spirit. Formally, karakia was performed by tohunga (healers) in all kinds of healing situations, and such knowledge was tribe-specific and never shared [74]. The tradition of karakia remained an essential part of Māori society, and today is performed in different settings.

### 4.3. Tikanga (Customs) and Whakaora (Healing)

Within this superordinate theme, two aspects were identified by the healers: first, the importance of establishing a relationship with the patient rather than focusing on the illness; second, the adoption of tikanga (customs) rongoā as a series of healing interventions.

Most healers shared that the core of the healing process is accepting the gift bestowed on them by the atua (gods), which allows them to understand better and read the environment they are in. Some mentioned that becoming a healer often involved processes of learning and discovery, mostly self-introspection to reconnect with self and understand how to negotiate their role as a healer. The starting point for most was to look inside themselves for answers.

*“Often, when somebody is unwell, I would feel whatever they have first in my own skin, you know… the quick I can make sense of it, better I can offer assistance.”*

(participant 8, female)

Rongoā practitioners mentioned that this enacted sensitivity and self-awareness are essential to maintain their balance and their own well-being and identity, ultimately contributing to their clients’ healing. A relationship would be formed between the healer and the patient to put the patients at ease and assist them in healing their lives. Questions about their whakapapa (genealogy), whenua (land) and whānau (extended family) would foster that relationship-building, one based on aroha (love and respect).

*“The first thing is to put the person at ease and get to know them…. so I can connect in with that whenua [land] and whakapapa [genealogy].”*

(participant 2, male)

*“Aroha [love and respect] is the most critical part of the healing process… oh yes, it needs to be with your patient...it is that love that helps with the healing.”*

(participant 3, female)

While rongoā is seen as a ‘people medicine’, caring for and healing people [51], many healers reported numerous examples of healing that have a physical, emotional and mental impact on patients. The most common healing interventions referred to by the rongoā healers included those at a spiritual level related to balancing energies, clearing fear and strengthening through belief [75], but also those at a physical level with visible body ailments, thus illustrating the relationship-based treatment and holist nature of healing:

*“So, I came here first, I stopped there dropped him off some rongoā pain relief, so by the time I got back there after here, he hadn’t chopped his leg off. We gauged it from Monday, by Wednesday he was fine, by Thursday it was still a bit tender, but he pushed it and by Friday he was running around. Saturday, he was going nightclubbing.”*

(participant 5, female)

*“I’ve got a two-year-old mokopuna [grandchild] that has had eczema since, I don’t know, the word goes, of which we actually have to use rongoā on him. Our children and their generation, they’re actually turning towards rongoā because they’ve studied to find out that mainstream medicine doesn’t work, and it isn’t fixing them, and they don’t trust it.”*

(participant 3, female)

Although Rongoā has mainly focused on spirituality as the primary source of treatment [43,48], the importance of mind, body and life force or spirit has become prominent over the last decades as a new underlying concept of holist healing. Nevertheless, the significance of knowledge, grounded in aroha (love and respect), was described as fundamental to assist health and well-being. Knowledge was seen as descending from ancestors and through to the current and future generations. Rongoā healers were perceived as the protectors of knowledge, which meant that their health and well-being depended on the presence (or not) of Māori knowledge to assist with the healing process of others.

### 4.4. Rākau (Plants)

The Rongoā healers extensively covered this superordinate theme. Rongoā rākau involves the use of the medicinal properties of plants and is often seen as a way to connect to the life force of the whenua (land). Most healers defended that rongoā is not about manufacturing a medicine; instead, it is about respecting nature and the mutual relationship between healers and plants [41]. The bark, leaves, roots, berries and branches are carefully harvested, removing only what is needed and ensuring that nature could continue to accommodate harvesting in the future. The healers describe the complexity of the process:

*“Rongoā is a different world, you know, it has its own process and generally takes a day… it starts with a karakia [chants] at home and… there’s more karakia [chants] while you walk the bush while you connect with the atua [gods] and with the rākau [trees, plants, herbs].”*

(participant 6, female)

*“It is about the exchange of mauri [life force] and whatumanawa [emotional] stuff… there’s a lot of hongi [pressing noses] with the rākau [plants] as they’ve their own stories, you know, they’re individuals like us.”*

(participant 8, female)

*“We get to know their [plants] whakapapa [genealogy], that’s the connection we want to have…cause everyone can boil stuff but it’s becoming tuned with the rākau [plants] that releases the full potential of it.”*

(participant 7, female)

Many healers corroborated the notion that the properties of rongoā healing extend beyond the physical and chemical properties to the connection of mauri (life force) of person, plant and healer, which are destined to be immersed together [76]. Plants are viewed as individual entities that are alive and can communicate their stories and genealogy to the healer. This relationship between healers and plants may be due to the belief of Māori healers in the ability of particular people to communicate with plants through their mauri (life force) [51,72]. In addition, when plants are taken, there is an acknowledgement of the spiritual world through karakia (chants) to give thanks. However, more importantly, there is also a recognition that the plants actively contribute to the healing process, even when preparing the remedies.

*“Depending on how connected with the taiao [environment] you are, you know which leaves to pick first…even from which tree…doesn’t matter the illness, the tuākana [elder siblings, referring to plants] always have the right answers.”*

(participant 1, male)

*“Even though the mauri [life force] of the rākau [plants] is being taken, you know, it returns to become one with people… helping them [people] in their journey.”*

(participant 4, female)

*“We make our own pani [spread] for our own rongoā and we utilise our native plants. For the pani, we use beeswax mainly. But, some whānau [extended family] are allergic to honey, therefore, they are allergic to beeswax. So, we use coconut oil as another source.”*

(participant 7, female)

While there is a strong association of Rongoā with the process of picking and preparing plants, other traditional healing techniques tend to be considered, like romiromi and mirimiri. The two latter techniques are unique forms of deep tissue massage and soft tissue massage, respectively, although they include some peculiar forms of treatment, including ‘spirit massage’ [25].

*“We’ve got marae clinics and kaumātua [eldery] days. We do mirimiri [soft tissue massage], whitiwhiti kōrero [advice/support] and honohono [deep tissue massage]. We also use native plants of our lands to make rongoā, ah, for different ailments.”*

(participant 5, female)

*“We do honohono, you know, energy work, so we don’t have to touch people at all… and whitiwhiti kōrero, which is about holistic well-being.”*

(participant 8, female)

*“We also do whānau mirimiri. We only touch the hands, the feet, and work the wairua [spirit].”*

(participant 2, male)

*“Mahi wheua, which is a realignment of the bones, but also gets rid of the uric acid in whānau [extended family], especially kaumātua [elders]. Um, it makes the blood flow so those crystals that crystallise around the bone don’t get to stay there.”*

(participant 6, female)

Through the different practices associated with Rongoā, reciprocal mutual communication between healers and plants is noticed. The entire process is performed to honour and respect the ancestry of the plants, continuing during the preparation of the herbal remedies. The same synergy is noticed when a healer works with soft tissue to improve body systems and energy flow and deep tissue alignment, pressure points, nerves and muscle tissue to induce change to the body and lead to positive change in mind and spirit.

### 4.5. Whenua (Land) and Whānau (Family)

The fifth superordinate theme discerned the importance of whenua (land) and whānau (family). Rongoā healers referred to the importance of land and its unique qualities and how that affects people and their health and well-being. Some mentioned that the land itself became the object of healing.

*“Healing is a holistic thing, you’re working with and for the whenua [land], once the whenua [land] is healed, then our people will feel better.”*

(participant 2, male)

*“The whenua [land] grounds our whānau [extended family], all whānau. You know, ‘cause some whānau [family] float around up in the sky. There’s a time for that, aye. I’m not saying that, you know, that’s not done, but there’s time for that, we need to anchor, and we anchor in our tipuna [ancestors] and whenua [land]. Especially us when we go to do mahi [work].”*

(participant 3, female)

Most healers defended the belief that a solid cultural identity greatly influences health and well-being and that identity is deeply embedded in whenua (land) [77]. In addition, participants mentioned that tikanga Māori (customs and traditions) acknowledges that individuals are inherently connected to one another, their surrounding landscape, and that one’s self is but a part of the more outstanding landscape, and the landscape is part of one’s being [58]. Many rongoā healers agreed that when the whenua (land), or the person, is damaged somehow, their mauri (life force) is also damaged or even lost. They agreed that their responsibility is to protect the whenua (land) and that the land can be healed, similar to healing a person [45,72].

Conversely, polluted and damaged land without mauri (life force) was expressed as a source of mamae (pain). There was a perception of illness of place, which in turn affected people’s health. For example, many participants were concerned with specific customs and traditions, like the burial of the placenta (whenua) in the ancestral lands of the tribe, often at the base of a marker tree, thereby linking the child to the tribal lands; or the sprinkling of water from a sacred stream onto a new-born child and dedicating the child to an atua (god). In contrast, the healers believed that such practices celebrated the primordial connection to Papatūānuku as the Mother Earth, and the whenua (the land/placenta) as the source of warmth, nourishment, and security; however, for them, the loss of land and population directly resulted in significant losses of cultural heritage. This is supported by similar research, which illustrates that identity is drawn from the connection to the land and that confiscation of territory was effectively stripping away Māori identity [78].

*“It’s amazing what water can do in the spiritual realm for us as iwi. We have a true belief in this awa [river]... that keeps us safe, within that tapu [sacred] realm of our Māori tikanga [customs and traditions].”*

(participant 1, male)

*“You know, if you were feeling not so good, you went back to your land... You took yourself and your troubles back to the land.”*

(participant 4, female)

This intricate connection to whenua (land) assists in explaining the relationship to place and between the healer and the wider environment. Most participants mentioned that land provided both physical and mental healing and was spoken to as a living entity; therefore, there was a reinforced commitment to kaitiakitanga (guardianship) of the land. Rongoā healers agreed that embracing traditional values enabled the reinstatement of mana (prestige/power) and whenua (land) of people and places. Nevertheless, many also agreed that rangatahi (youth) and tamariki (children) play an essential role in maintaining and protecting the whenua (land) as well as fostering rongoā mātauranga (knowledge) and tikanga (customs) because of their potential for learning and absorbing knowledge.

*“Our children are actually a bit more close to rongoā than we are. I do find that it’s taken time.”*

(participant 8, female)

*“Cause the kids go, ‘aunty, we’ve seen that stuff work.’ It ain’t my stuff, it’s our stuff. And as long as I’m picking those leaves from your whenua [land], it’s your stuff.”*

(participant 5, female)

*“So, really, these things that we have learned, to reach a young person, you’ve got to come down to their level, not expect them to come up to you. You want to reach your mokopuna [grandchildren] or whoever and put it across and make it exciting. Who the hell wants to listen to an old person? Oh, please.”*

(participant 6, female)

Whānau (family) is an important aspect of Māori culture yet has not been noted as a crucial aspect of rongoā. Therefore, the intersection of whānau (family), whenua (land) and rongoā is unique. The notion that healing transverses well beyond caring for the landscape and its conservation, relating them to peoples’ inner self and being (the mauri or life force). Healing is an active process conceived from the whenua (land), drawing from mātauranga and tikanga (knowledge and customs) and whakapapa (genealogy) by a healer through a myriad of rongoā practices, stimulating the hinengaro (mind), tinana (body) and wairua (spirit) to heal itself and valuing the aroha (love and respect) as a critical factor for its fulfilment.

## 5. Discussion

This study provided insights into the connection of landscape and healing through RM, adding to the research in this area. It demonstrated the importance of wairua (spirit) and tinana (body) in improving our physical, mental, spiritual and communal health and well-being. To accomplish this, the connection to whenua (land), whakapapa (genealogy), tīpuna (ancestors) and rākau (plants) was a critical aspect in the relationship between healers and patients. 

Māori have always held an expansive view of knowledge with a depth of understanding derived from both intellectual and spiritual pursuits [79]. While some of the results concerning spirit, body and mind are consistent with previous research [19,25,30], emphasis is placed on the landscape as the mediator. This supports the notion that the whenua (land) is accepted as the arena where the connection between the healer and tīpuna (ancestors) is established and the grounding place to engage with the whakapapa (genealogy) of the patient and the mauri (life force) of the rākau (plants). This conception of whenua (land) expands the existing ecological and biophysical understandings to accommodate the place-specific cultural and spiritual significance. As living with nature implies the guardianship of both land and people, places are seen as sacred [80]. In this manner, health, healing and well-being are directly associated with a site or place.

Creating places where people can connect with the whenua (land) is crucial for developing health and well-being. While the natural environment in the past has provided Māori with access to resources through forests, waterways and wetlands, the current danger is of losing traditional customs, narratives and healing practices and increasingly turning to Western therapy methods, independent of the land. Possessing a solid cultural identity goes beyond knowing ancestral heritage; it considers the ecological, economic and social contexts, which provide a holistic understanding that underpins a positive health outcome [81]. The resultant bond formed from the landscape and the healing process explains why certain places or situations are perceived as therapeutic for rongoā practitioners. In this regard, the principles of RM apply equally to healing the land as to healing those who are part of the land.

**Figure 4:**
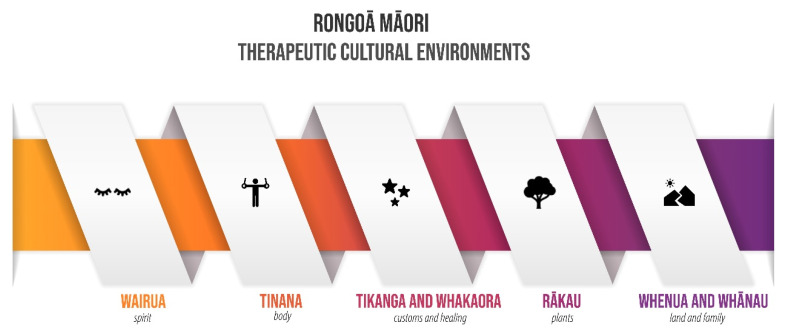
Elements that are part of therapeutic cultural environments based on Rongoā healers.

The link between whenua (land) and the health of Indigenous people has been acknowledged internationally [9,27,82,83,84]. However, few studies explore the connection between the landscape and the health of people culturally associated with it [17,85,86], mainly described as ‘therapeutic cultural landscapes’. By understanding therapeutic cultural landscapes, key concepts can be amalgamated and lead to meaningful and reflective places. It was evident for rongoā practitioners that the ideals inherent in these therapeutic cultural landscapes should not be seen as separate entities but part of a broader holistic system that caters to people’s senses, emotions, and values. People’s interactions with the land provide a spiritual connection; those connections cannot be gained through disenfranchised minds. The land cannot be healed nor managed with respect if the connections to it are jeopardised (Figure 4).

Focusing on therapeutic needs and values can combat and shape the way people and nature work and live together, similar to traditional ways. For Māori, the traditions of tikanga (customs) are vital as they are the customary ways of doing and acting. For non-Māori, cultural and therapeutic landscapes can be seen as a part of modern ways of living [10]. In addition, many rongoā healers referred to the need to re-establish the balance on the landscape, allowing the growth and harvest of rākau (plants) and preserving the tikanga (customs) around rongoā. Similar concerns are shared in different fields of practice, including the built environment disciplines. For many researchers [84,87], a sense of coherence can be achieved through a salutogenic approach of connecting the individual, the group and the broader environment [88]. A strong sense of belonging and coherence is reported when spaces are designed to foster a healthy balance of mind, body and spirit [89,90]. By restoring the land and acknowledging the significance of the site, we can aid the health of those drawing resources from the site. Such findings also align with other theoretical frameworks, such as topophilia (place attachment) and biophilia (innate human connection to nature). The more exposure people have and the more profound their immersion within the natural environment, the deeper their understanding will be, allowing them to heal themselves through this process.

This research acknowledges that the social and ecological difficulties faced by those practising rongoā are far more complex and multifaceted than the scope of this study can convey. Traditional healing has had to and will continue to adapt to a changing environment with pressures from urbanisation, ecological impacts and social change [41]. However, a re-emergence in the interest of rongoā offers opportunities for iwi (tribes), hapū (sub-tribes) and whānau (family) to embrace their cultural identity and support the sustainable practice of rongoā into the broader community. The importance of maintaining traditional values and beliefs within a contemporary context is fundamental as it permits to better comprehend the relationship between people and landscape and how the landscape can function holistically. For some, it is not “not only culturally appropriate, but it is vital to Indigenous health, healing, and well-being in a new and changing world” [30]. Moreover, they should conserve and promote cultural heritage and build on natural features to inspire a deep connection to place and provide diverse habitats for appropriate species to enhance biodiversity and create safe and healthy places for connecting with others and recreation and mental relaxation.

## 6. Conclusions

In a world of constant development, rongoā healers are constantly adapting their methods as new opportunities and changes in the environmental and social climate occur. This remains as true today as it was in the past. When colonial settlers first arrived on Aotearoa/New Zealand’s shores, they brought new technology and medicines and new diseases. Settlers also brought exotic species that impacted native ecosystems and contributed to the decline of mauri (life force). Māori were quick to adopt new ways to prepare and store rongoā. It is widely accepted that colonisation profoundly affected the use and application of rongoā for Māori. The whenua (land) reminds Māori of how difficult it is to find remnants of native vegetation that once were part of Papatūānuku (Mother Earth), profoundly affecting their own sense of identity and connection. Despite the forced suppression and the stigmatisation of rongoā throughout the decades, the knowledge continued to be passed down through the generations and is not entirely lost today.

This article aimed to provide insight into how rongoā healers connect to the whenua (landscape) and which aspects are critical to attaining while addressing therapeutic cultural environments for healing, health and well-being. It maintains that cultural and therapeutic landscapes can be seen as part of modern living rather than relics of bygone times. Often conventional well-being models are rigid in approach; however, by understanding the holistic interrelation of wairua (spirit) and tinana (body) in improving physical, mental, spiritual and communal health and well-being, we better comprehend the extent of the relationship between healers and patients. The research findings show how rongoā is underpinned by a complex set of cultural values and beliefs, drawing from the connection to whenua (land), whakapapa (genealogy), tīpuna (ancestors) and rākau (plants). Incorporating such constructs can facilitate a restorative and therapeutic landscape and offer new opportunities for living with nature and supporting health and well-being. Landscape Architecture can enable place-specific understandings of health and wellbeing for better land management and encourage better practices associated with native planting, promotion of chemical-free farming, expansion of native forests, decontamination of the water table, and protection of sacred sites.

When engaging with sensitive information surrounding cultural safety and Indigenous knowledge, working with iwi (tribes) is fundamental to understanding practitioners’ values and concerns. Only by engaging with the historical impacts and modern barriers can we start to look to the future of rongoā and begin to unpack what the future might look like and work towards a respectful and beneficial future. Understanding the complex layers that make up our therapeutic cultural landscape and the deeper connection required to practise rongoā safely and with respect can enable people to feel a sense of security and safety with the place. These feelings of belonging allow for making whakapapa (genealogy) with the landscape while healing self and place for a more resilient future. For rongoā healers, the whole environment is seen as therapeutic and is another layer developed upon the cultural and ancestral landscapes. In this manner, the concepts of wairua (spirit), tinana (body), tikanga and whakaora (customs and healing), rākau (plants), whenua (landscape) and whānau (family) incorporate culture in a symbiotic model where caring landscapes are placed within the framework of landscape authenticity and caring people heal the landscape that nurtures them.

## Figures and Tables

**Table 1:** Interview questions.

Open-Ended Questions	Dichotomous Questions (Yes or No)
Can you explain what rongoā means to you? How does it work?	Do you live in your rohe (tribal boundaries)?
What is the relationship between healing and mātauranga (knowledge) and tikanga (customs) Māori? What is the relationship with the wider landscape/nature?	Do you live in the city?
How could we use the landscape to maintain our health and wellbeing?	Do whānau (family) live nearby?
Are there places that have special meaning for you? What are the qualities of the place/s that mean it is good to harvest from?	Do you provide help to whānau (family)?
What do you consider when practicing rongoā?	Do you have mokopuna (grandchildren) or tamariki (children)?
How do you see rongoā and other modes of traditional healing being passed on? How do you make sure that rongoā is around for future generations?	Do you have links with your local marae (Māori meeting places) or haukāinga (local people of a marae)?
What kind of relationship should Māori traditional healing have with mainstream health services?	Do you collect any plants, berries, other materials for rongoā?
What are some of the ways in which you ensure your practice is protected?	Are there ngahere (forest), rākau (plants) or awa/moana (river/lake) near you that you use?

**Table 2:** Phases of thematic analysis in IPA (adapted from adapted from [65,66,67]).

Step	Description
1. Data Familiarisation	Transcription, reading and re-reading of data, initial ideas
2. Codes	Identification and organisation of the data into overarching codes
3. Themes	Interpretation of data and collation of codes into potential themes based on patterns and commonalities found
4. Review	Identified themes and sub-themes will be rechecked and refined in relation to the coded extracts and then the entire data set
5. Define and Name	Revision and refinement of the higher themes in relation to lower themes (and vice-versa) to ensure consistency, generating clear definitions and names for each theme
6. Results	Report on findings. A compelling narrative where the themes are weaved together with data extracts is required

## Data Availability

Data presented in this study are available on request from the corresponding author. The data are not publicly available due to ethical and privacy restrictions.
